# Efficacy and Safety of Platelet-Rich Plasma Versus Topical Minoxidil in Androgenetic Alopecia: A Systematic Review

**DOI:** 10.7759/cureus.115244

**Published:** 2026-08-26

**Authors:** Angel I Garcia Prado, Domenica P Ramos Cristiansen, Dinorah A Robles Rangel, Reiner S Moreno Hidalgo

**Affiliations:** 1 Department of General Medicine, Universidad Católica de Santiago de Guayaquil, Guayaquil, ECU; 2 Department of General Medicine, Universidad Anáhuac México, Huixquilucan, MEX; 3 Department of General Medicine, Escuela Superior Politécnica de Chimborazo, Riobamba, ECU

**Keywords:** alopecia, hair, minoxidil, platelet-rich plasma, treatment outcome

## Abstract

Choosing between single-agent and combined therapies for androgenetic alopecia (AGA) remains a frequent clinical challenge, particularly when comparing topical options against autologous treatments. This study compares how 5% topical minoxidil and autologous platelet-rich plasma (PRP) perform against each other alone and in combination, focusing on efficacy, side effects, and patient satisfaction. The search covered the Cochrane Central Register of Controlled Trials (CENTRAL), Medical Literature Analysis and Retrieval System Online (MEDLINE), PubMed, Scopus, Latin American and Caribbean Health Sciences Literature (LILACS), and Scientific Electronic Library Online (SciELO) up to May 2026. We targeted prospective randomized controlled trials (RCTs) comparing or combining minoxidil 5% with PRP in adult cohorts (≥18 years). Quality assessment relied on Cochrane Risk of Bias 2 (RoB 2). Four trials enrolling 226 patients qualified for analysis. Monotherapies displayed distinct clinical patterns. Topical 5% minoxidil generated steady hair shaft thickness over extended follow-up. In contrast, monthly PRP injections induced rapid initial hair pull test conversion by week 12. Combined administration showed more favorable clinical outcomes compared to individual monotherapy arms across evaluated endpoints, with individual trials reporting up to 66.7% hair diameter gains, higher trichoscopic hair counts, and patient satisfaction rates reaching up to 90%. Complications remained local and minor, presenting as mild scalp itching with minoxidil and transient post-injection soreness with PRP. Marked variation in PRP preparation methods, spin protocols, and injected volumes prevented quantitative pooling. Studies evaluated predominantly male cohorts during short six-month tracking windows. Single-agent regimens provide partial responses without clear overall dominance. Adding monthly PRP injections to daily 5% minoxidil demonstrates promising comparative efficacy for vertex density restoration, though further standardized trials are warranted.

## Introduction and background

Global epidemiological data confirms that androgenetic alopecia (AGA) remains the primary driver of progressive hair thinning in dermatological consultations [1]. This condition triggers a systematic miniaturization process where hair follicles, under strict androgen control, transform thick terminal fibers into thin vellus hair [2]. Research on non-scarring alopecias consistently highlights how dihydrotestosterone attaches to receptors in the dermal papilla, disrupting normal cycling and shortening the growth window [3]. Beyond the structural impact on the scalp, the cosmetic changes often induce severe emotional stress across clinical severity stages, typically graded using the Hamilton-Norwood scale for male-pattern and the Ludwig scale for female-pattern alopecia, forcing clinicians to search for reliable, standard interventions [4].

For several decades, patients have relied on topical minoxidil 5% as their primary defense to preserve hair density [4]. This chemical compound works by opening potassium channels, encouraging local blood flow, and lengthening the anagen phase [4]. Even so, messy daily routines and scalp irritation frequently hurt long-term patient compliance [5]. Autologous platelet-rich plasma (PRP) emerged to address these limitations, shifting the therapeutic approach from daily non-invasive self-application to periodic, physician-administered intradermal injections that deploy biological growth factors directly to the hair roots to kickstart papilla cell division [6]. Yet, current clinical trials present conflicting conclusions when deciding whether standalone plasma injections deliver a better safety profile or higher hair counts than standard topical choices [7].

Practicing dermatologists face a cluttered landscape because existing comparative trials utilize highly diverse centrifugation speeds, manual or automated tools, and mismatched follow-up dates. This structural variation makes it difficult to pinpoint exactly when combined protocols offer a true advantage over monotherapy.

To address this uncertainty, this systematic review specifically synthesizes prospective randomized controlled trials (RCTs) published between 2021 and 2026 comparing PRP against 5% topical minoxidil, both as monotherapies and in combination. By focusing on recent trials, this review uniquely evaluates modern standardized PRP preparation protocols, contemporary trichoscopic endpoint metrics (such as hair shaft diameter gains), and patient satisfaction outcomes to provide an updated, non-fragmented breakdown of their comparative efficacy and safety profile

## Review

Methods

*Study Registration and Protocol* 

This systematic review was prospectively registered in the International Prospective Register of Systematic Reviews (PROSPERO) under registration number CRD420261447451 [8].

Study Criteria and Eligibility

The methodological design followed the operational steps mandated by the Preferred Reporting Items for Systematic Reviews and Meta-Analyses (PRISMA) 2020 statement [9]. To filter the literature effectively, selection parameters were organized around a dynamic Population, Intervention, Comparator, and Outcomes (PICO) structure [10]:

Population (P): Clinical trial cohorts presenting an established diagnosis of AGA were included, focusing exclusively on individuals aged 18 years or older, with no restrictions regarding participant sex. Conversely, criteria excluded any baseline presenting with scarring forms of hair loss, chronic telogen effluvium, or alopecia stemming from active oncological chemotherapy or unstable systemic pathology.

Intervention (I): The core clinical track demanded the use of autologous PRP treatments, whether delivered as a monotherapy or combined with topical minoxidil regimens. Studies evaluating co-interventions such as microneedling, exosomes, or systemic agents without an isolated PRP vs. minoxidil comparative arm were excluded.

Comparator (C): Comparative arms had to evaluate topical minoxidil 5% solutions or foams, alongside relevant vehicle placebos or monotherapy comparators. Investigations dealing solely with non-5% concentrations or oral systemic medications (e.g., oral minoxidil, finasteride, or dutasteride monotherapies) were excluded.

Outcomes (O): Matching studies had to provide objective metrics for hair density shifts, hair count variations, or absolute changes in hair shaft diameter while documenting secondary safety parameters such as scalp irritation or regional pain.

To maintain real clinical relevance for modern dermatology, the search was restricted entirely to prospective designs, RCTs, published between 2021 and 2026. This restricted timeframe was deliberately defined to evaluate contemporary trials reflecting modern standardized double-spin PRP protocols and standardized quantitative trichoscopic metrics, thereby minimizing clinical heterogeneity from obsolete preparation techniques. This timeframe intentionally left out non-randomized comparative studies, case series (n < 5), personal editorials, letters to the journal editor, and conference presentations missing complete numerical results.

Information Sources and Search Strategy

The literature retrieval involved systematic queries across PubMed, Medical Literature Analysis and Retrieval System Online (MEDLINE), Scopus, Scientific Electronic Library Online (SciELO), Latin American and Caribbean Health Sciences Literature (LILACS), and the Cochrane Central Register of Controlled Trials (CENTRAL). Our search approach combined text keywords and official medical subject descriptors (MeSH terms for PubMed/MEDLINE) through logical Boolean connectors. The implemented search sequence was ("androgenetic alopecia" OR "pattern hair loss") AND ("platelet-rich plasma" OR "PRP") AND ("topical minoxidil" OR "minoxidil"). Full-text availability was required to ensure thorough data extraction.

The detailed database-specific search strategies, including controlled vocabularies (MeSH), field tags, filters, search dates, and exact Boolean operators for all searched databases, are provided in Table 1.

**Table 1:** Detailed database-specific search strategies and query strings Database-specific search strategies include controlled vocabularies, field tags, Boolean operators, and applied filters used to identify eligible studies published up to May 2026. CENTRAL: Cochrane Central Register of Controlled Trials; MeSH: Medical Subject Headings; LILACS: Latin American and Caribbean Health Sciences Literature; SciELO: Scientific Electronic Library Online

Database	Search strategy/syntax	Filters/limits	Search date
PubMed / MEDLINE	("Alopecia, Male"/ethnology OR "Alopecia, Male"[Mesh] OR "androgenetic alopecia"[Title/Abstract] OR "pattern hair loss"[Title/Abstract]) AND ("Platelet-Rich Plasma"[Mesh] OR "platelet-rich plasma"[Title/Abstract] OR "PRP"[Title/Abstract]) AND ("Minoxidil"[Mesh] OR "topical minoxidil"[Title/Abstract] OR "minoxidil"[Title/Abstract])	Humans, Clinical Trial, Randomized Controlled Trial, English, Spanish, Portuguese	May
CENTRAL	(#1 MeSH descriptor: [Alopecia] explode all trees OR "androgenetic alopecia":ti,ab,kw OR "pattern hair loss":ti,ab,kw) AND (#2 MeSH descriptor: [Platelet-Rich Plasma] explode all trees OR "platelet-rich plasma":ti,ab,kw OR "PRP":ti,ab,kw) AND (#3 MeSH descriptor: [Minoxidil] explode all trees OR "topical minoxidil":ti,ab,kw OR "minoxidil":ti,ab,kw)	Trials	May 2026
Scopus	TITLE-ABS-KEY ( ( "androgenetic alopecia" OR "pattern hair loss" ) AND ( "platelet-rich plasma" OR "PRP" ) AND ( "topical minoxidil" OR "minoxidil" ) )	Article, Article in Press, English, Spanish, Portuguese	May 2026
SciELO	("androgenetic alopecia" OR "pattern hair loss") AND ("platelet-rich plasma" OR "PRP") AND ("topical minoxidil" OR "minoxidil")	All Indexes	May 2026
LILACS	("androgenetic alopecia" OR "calvicie") AND ("platelet-rich plasma" OR "plasma rico en plaquetas") AND ("minoxidil")	Humans	May 2026

Study Selection and Data Extraction

Two independent reviewers screened titles and abstracts retrieved from the electronic search according to predefined eligibility criteria. Full-text articles of potentially eligible studies were subsequently retrieved and assessed independently for inclusion. Any disagreements between reviewers regarding study eligibility were resolved through discussion or consultation with a third author. Data extraction was conducted independently using a standardized data extraction form to capture study characteristics, baseline participant demographics, PRP and minoxidil intervention protocols, primary efficacy outcomes (hair density and shaft diameter), and reported adverse events.

Risk of Bias Assessment

Methodological quality and vulnerability to systematic errors across all included RCTs were independently evaluated using the Cochrane Risk of Bias 2 (RoB 2) tool [11]. Evaluations were conducted at the outcome level, specifically differentiating primary objective endpoints (trichoscopic hair density and shaft diameter) from secondary subjective endpoints (patient self-assessment and physician global impression). Objective outcomes generally maintained a lower risk of bias regarding measurement, whereas subjective endpoints in unblinded trials presented a higher risk of performance and detection biases. Five analytical domains were examined: randomization process, deviations from intended interventions, missing outcome data, measurement of the outcome, and selection of the reported result. Discrepancies between evaluators were resolved through joint re-examination of trial protocols and consensus. Risk of bias plots were generated using the RobVis web application. To evaluate the overall certainty of evidence for primary outcomes, the Grading of Recommendations Assessment, Development, and Evaluation (GRADE) approach was applied across domains of risk of bias, inconsistency, indirectness, imprecision, and publication bias.

Data Synthesis

Clinical and statistical comparability across included RCTs was systematically assessed prior to quantitative synthesis. Although random-effects pooling was considered, a formal meta-analysis was not performed due to critical clinical heterogeneity in PRP protocols (differences in centrifugation speeds, spin numbers, and activation agents) and non-standardized outcome reporting metrics (varying trichoscopic measurement units and follow-up timelines). Consequently, a structured narrative synthesis was conducted to prevent misleading pooled estimates. Instead, a structured narrative synthesis was conducted, grouping findings by monotherapy comparative efficacy, combined protocol synergies, and safety/adverse event profiles.

Results

Study Selection and Screening Flow

**Figure 1:**
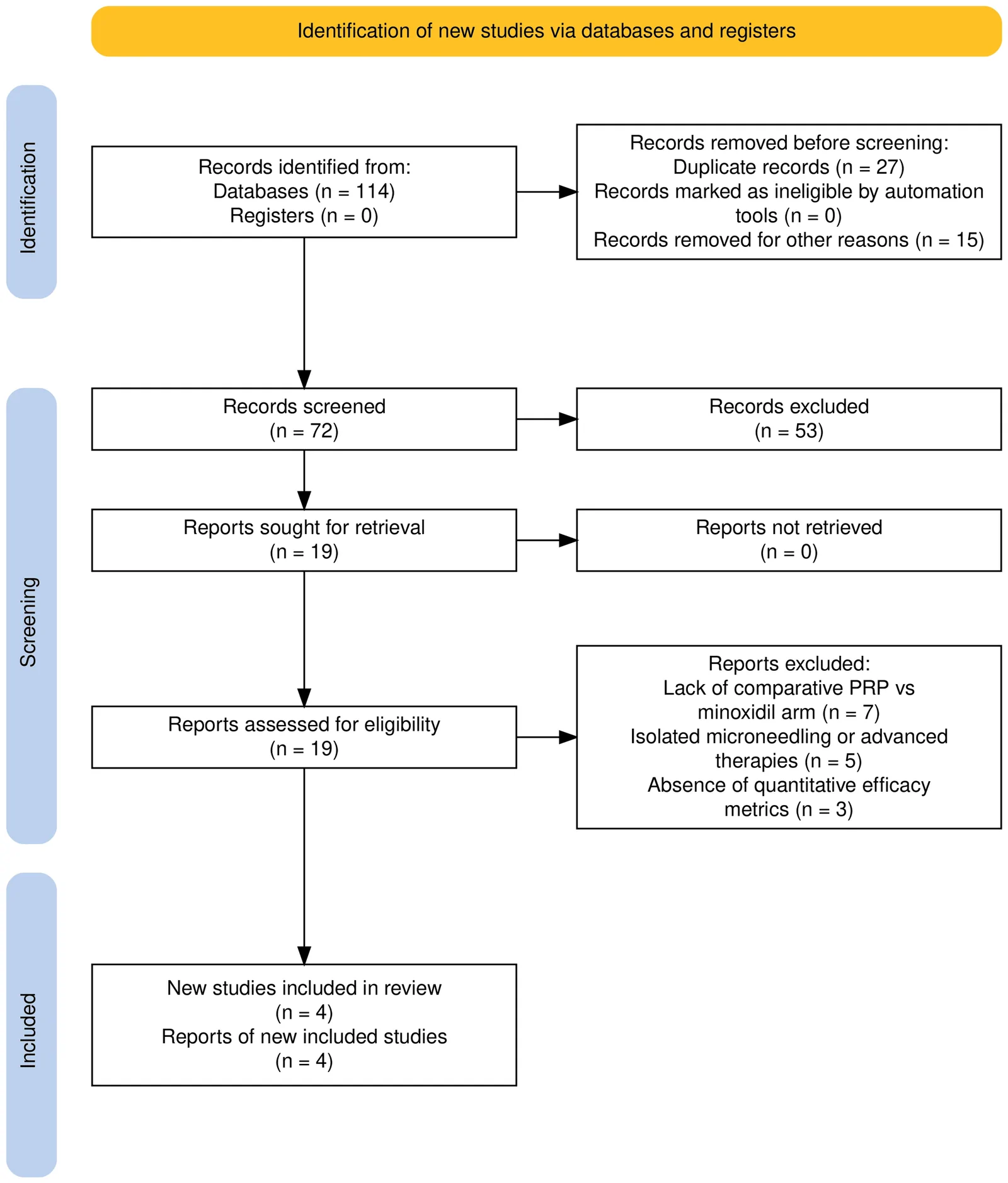
PRISMA 2020 flow diagram for the systematic review process PRISMA 2020 flow diagram for the systematic review process, detailing study identification, screening, eligibility, and inclusion. CENTRAL: Cochrane Central Register of Controlled Trials; LILACS: Latin American and Caribbean Health Sciences Literature; SciELO: Scientific Electronic Library Online; PRP: platelet-rich plasma; PRISMA: Preferred Reporting Items for Systematic Reviews and Meta-Analyses

Our electronic searches across medical databases through May 2026 initially returned 114 entries. After dropping 27 duplicates, we screened out 15 non-peer-reviewed items, mostly editor letters or brief comments. That left 72 citations. Evaluating titles and abstracts led us to exclude 53 studies, mainly off-target research focusing on non-AGA, oral-only regimens, or animal models. We retrieved 19 full-text manuscripts. During full-text eligibility screening, 15 reports were excluded for failing to meet predefined inclusion criteria: seven lacked direct PRP versus minoxidil comparative arms, five evaluated adjunctive procedures (e.g., microneedling) without an isolated PRP monotherapy group, and three omitted required quantitative primary endpoints. Four prospective randomized trials met every selection requirement (Figure 1) [12-15].

Methodological and Population Characteristics

Table 2 outlines baseline setups for these four included RCTs. Tracking durations spanned from 12 weeks up to six months across a combined cohort of 226 patients with active AGA. All four trials relied on prospective, randomized setups, enrolling mostly male subjects presenting with vertex or frontal thinning, alongside targeted female subgroups in specific studies [12-15].

**Table 2:** Main methodological characteristics and intervention regimens of the included clinical trials AGA: androgenetic alopecia; PRP: platelet-rich plasma; RCT: randomized controlled trial

Study (author, year)	Study design	Sample size	Population	Technical arms	Intervention regimens
Balasundaram et al., 2023 [12]	Open-label RCT	N = 64	Males (20–50 years old) with AGA grade III-IV	Group 1: Topical Minoxidil 5%; Group 2: Non-activated autologous PRP	Group 1: Daily for 6 months; Group 2: Monthly injections for 3 months
Wei et al., 2023 [13]	Double-blind RCT	N = 30	Male patients with mild to moderate AGA	Group A (PRP+M): PRP prepared by automatic blood cell separator combined with topical 5% minoxidil; Group B (PRP+P): PRP combined with topical placebo for 3 months	Twice daily (minoxidil/placebo) for 3 months
Shah et al., 2023 [14]	RCT	N = 72	Patients (60 males, 12 females; aged 20–40 years) with AGA (Stages I–VII)	Group A (PRP): Monthly autologous PRP injections; Group B (Minoxidil): Topical Minoxidil 5% (1 ml)	Group A: Monthly for 3 months; Group B: Twice daily for 3 months
Annamreddy et al., 2024 [15]	Single-center randomized prospective interventional study	N = 60	Untreated patients with AGA (52 males, 8 females; age range: 18–38 years; mean age: 25.2 ± 4.08).	Group I (PRP+M): Topical Minoxidil 5% (1 mL) + Intradermal PRP (3–4 mL); Group II (Minoxidil): Topical 5% minoxidil (1 mL); Group III (PRP): Intradermal PRP injections (3–4 mL)	Group I: Minoxidil twice daily + Monthly PRP for 6 months; Group II: Twice daily for 6 months; Group III: Monthly for 6 months

Risk of Bias and Quality Assessment Results

Evaluation using the Cochrane RoB 2 tool demonstrated variable methodological quality across the four included RCTs [12-15]. Overall risk of bias was low to moderate for primary objective outcomes (trichoscopic hair density and shaft diameter), whereas subjective endpoints (patient satisfaction and physician global impression) presented higher vulnerability to performance and detection biases in unblinded designs.

Specifically, only Wei et al. (2023) demonstrated a low risk of bias across all domains, owing to solid allocation concealment and double-blind controls [13]. Balasundaram et al. (2023) raised some concerns; although missing data and reporting bias risks were low, its open-label design introduced bias in intervention deviations and outcome assessment [12]. Two trials were judged as high risk overall: Shah et al. (2023) [14] and Annamreddy et al. (2024) [15]. Shah et al. suffered from concerns across multiple areas, including randomization, protocol deviations, and outcome measurements [14]. For Annamreddy et al., the high risk was mainly driven by Domain 2 issues related to an unblinded protocol paired with subjective endpoint measures, alongside unclear sequence generation in Domain 1 [15].

**Figure 2:**
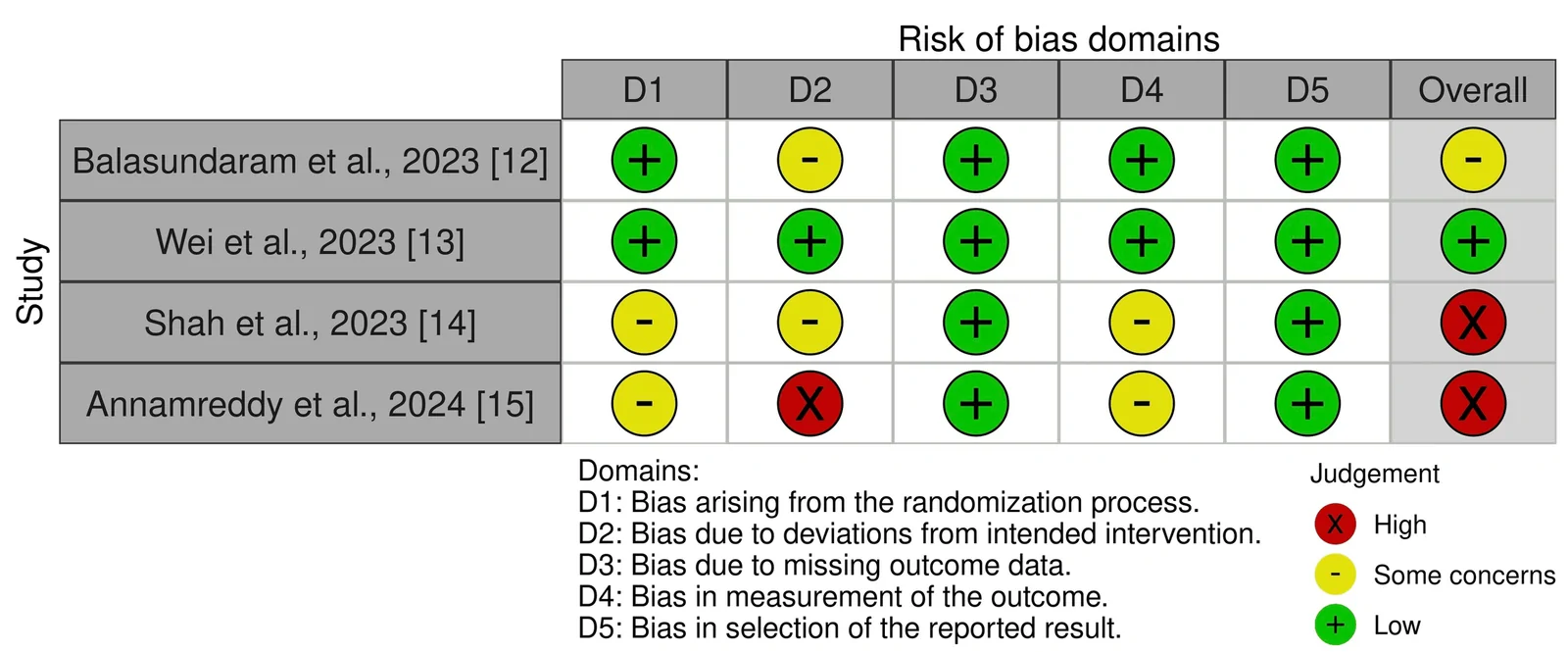
Risk of bias traffic-light plot for included randomized controlled trials Risk of bias assessment generated using the Cochrane RoB 2 tool via the robvis application. Domains: D1 (randomization process); D2 (deviations from intended interventions); D3 (missing outcome data); D4 (measurement of the outcome); D5 (selection of the reported result). [12-15]

Domain-specific assessments and overall risk of bias summaries are illustrated in Table 3 and Figure 2. According to the GRADE approach, the certainty of evidence for hair density and shaft diameter improvements was rated as moderate due to minor imprecision and methodological heterogeneity across PRP centrifugation protocols. Because two of the four included RCTs exhibit a high overall risk of bias, the comparative findings synthesized across this review are interpreted with appropriate methodological caution throughout the discussion.

**Table 3:** Risk of bias assessment for included randomized controlled trials (Cochrane RoB 2) Note: Assessment performed using the Cochrane Risk of Bias 2 (RoB 2) tool for randomized controlled trials. Domains: D1: bias arising from the randomization process; D2: bias due to deviations from intended interventions; D3: bias due to missing outcome data; D4: bias in measurement of the outcome; D5: bias in selection of the reported result. Overall risk: categorized as low risk (low risk across all domains), some concerns (at least one domain with some concerns, but no high risk in any domain), or high risk (high risk in at least one domain or multiple domains with some concerns).

Study (year)	D1: Randomization process	D2: Deviations from intended interventions	D3: Missing outcome data	D4: Measurement of the outcome	D5: Selection of the reported result	Overall risk of bias
Balasundaram et al., 2023 [12]	Low	Some concerns	Low	Low	Low	Some concerns
Wei et al., 2023 [13]	Low	Low	Low	Low	Low	Low risk
Shah et al., 2023 [14]	Some concerns	Some concerns	Low	Some concerns	Low	High risk
Annamreddy et al., 2024 [15]	Some concerns	High risk	Low	Some concerns	Low	High risk

Comparative Monotherapy: Standalone PRP Versus Minoxidil 5%

Comparative outcomes between single-agent PRP injections and daily 5% topical minoxidil varied by endpoint. In Balasundaram et al., objective hair density gains appeared in both arms by week 12 (p < 0.05) [12]. By week 24, overall response rates showed no statistical gap between minoxidil (56%) and monthly non-activated PRP (38%; p = 0.124). Patient-assessed hair texture changes, however, clearly favored minoxidil (p = 0.029).

Shah et al. observed faster initial responses with PRP alone [14]. By week 12, negative hair pull test rates hit 91.7% in the PRP group versus 69.4% in minoxidil controls (p = 0.017). This gap widened in early vertex loss (Stage II), reaching 92.9% for PRP against 46.7% for minoxidil (p = 0.007). Conversely, Annamreddy et al. recorded a 50.0% increase in six-month hair shaft diameter with PRP, outperforming minoxidil alone (35.20%; p = 0.0004), despite comparable overall alopecia grade improvements across both single-agent groups [15].

Synergistic Combination and Multimodal Strategies

Combining PRP infiltrations with topical minoxidil showed more favorable outcomes across several measured endpoints compared to single-agent approaches. Wei et al. tested a three-month combination of daily 5% minoxidil plus automated PRP, finding significant hair count gains (p < 0.05), whereas the difference in hair density between groups did not reach statistical significance (p = 0.26), alongside higher satisfaction (86% vs 73%) [13].

Annamreddy et al. confirmed these additive benefits over six months [15]. Patients receiving combined monthly PRP and daily minoxidil gained 66.70% in hair shaft diameter (p = 0.0004). Marked physician-rated improvements appeared in 85% of dual-therapy subjects, and 90% expressed high personal satisfaction, outperforming both single-agent comparisons. However, these findings derive from a single trial evaluated as having a high overall risk of bias (RoB 2) and should therefore be interpreted with caution until validated by larger, low-bias trials.

Safety Profile and Adverse Events

Safety profiles reported across three of the four RCTs showed mild-to-moderate, localized reactions without systemic complications, whereas Shah et al. [14] did not report adverse event metrics (Table 4). Topical minoxidil prompted mainly local scalp pruritus, mild erythema, or flaking. Injections of autologous PRP produced transient pain, burning, and post-procedure headaches. In Balasundaram et al., injection discomfort caused four patients (6.25%) to drop out of manual PRP therapy [12]. Combined protocols recorded strong overall adherence with zero patient dropouts.

**Table 4:** Clinical outcomes, efficacy variables, and safety profiles of the analyzed studies Statistically significant difference (p < 0.05). AGA: androgenetic alopecia; PRP: platelet-rich plasma; RCT: randomized controlled trial

Study (author, year)	Follow-up	Evaluation metrics / Outcomes	Key findings	Documented adverse events	Withdrawals
Balasundaram et al., 2023 [12]	Follow-up: 24 weeks	Global photography (week 24); phototrichogram (week 12); patient satisfaction; adverse events	At week 24, global response was 56% for Minoxidil vs. 38% for PRP (p = 0.124). Both groups showed significant density improvements at week 12 (p < 0.05). Patient satisfaction regarding hair texture was higher for Minoxidil (p = 0.029).	Minoxidil 37% (*n* = 12/32) vs. PRP 53% (*n* = 17/32) (p = 0.21). Minoxidil: mild headache, scalp pruritus, 1 contact dermatitis. PRP: transient post-injection pain (n = 17).	4 patients withdrew due to pain.
Wei et al., 2023 [13]	Follow-up: 4 weeks after last injection (Total: 4 months)	Trichoscopic evaluation (20x); global photography; patient questionnaire.	Both groups showed significant increases in hair density and count post-treatment (p < 0.05). Density increase was more pronounced in Group A than Group B, but without statistical significance (p = 0.26). Group A satisfaction was 86% vs. 73%.	No serious events. Both arms reported transient pain, burning sensations, localized bleeding, and erythema (alleviated with ice). One patient experienced mild dizziness and nausea during PRP injection.	None reported.
Shah et al., 2023 [14]	Follow-up: 12 weeks	Hair pull test (negative if <4/50 pulled); baseline hair density calculation.	Baseline density: 83.50 ± 39.63 cm^2^ (PRP) vs. 88.94 ± 36.09 cm^2 ^(Minoxidil). At week 12, the negative hair pull test rate was higher in PRP (91.7%) vs. Minoxidil (69.4%) (p = 0.017). Stage II patients achieved 92.9% vs. 46.7% (p = 0.007).	Not reported in the analyzed text.	None reported.
Annamreddy et al., 2024 [15]	Follow-up: 6 months (assessed at baseline, 3, and 6 months)	Trichoscopy (hair shaft diameter, single hair follicular units, yellow dots); AGA grade staging (Hamilton-Norwood/Ludwig); physician scale of assessment; patient self-assessment; global clinical photography.	Alopecia Grade Improvement (p = 0.0001): 55% in Group I, 40% in Group III, and 25% in Group II. Trichoscopic Hair Shaft Diameter Improvement (p = 0.0004): 66.70% in Group I, 50.00% in Group III, and 35.20% in Group II. Physician Scale (>50% improvement): 85% in Group I, 70% in Group III, and 55% in Group II. Patient Self-Assessment Improvement: 90% in Group I, 70% in Group III, and 50% in Group II.	Group I (PRP+M): 10 patients (50%) reported side effects; most common were headache and post-injection pain (PRP), followed by itching, irritation, and hypertrichosis (Minoxidil). Group II (Minoxidil): 13 patients (65%) experienced side effects; the most common were scalp irritation, followed by itching and hypertrichosis. Group III (PRP): 10 patients (50%) reported side effects; the main complaint was post-injection pain, followed by headache.	No dropouts or withdrawals reported.

Discussion

Therapeutic choices in AGA are shifting as clinical data contrasts standard topical treatments against autologous biostimulation [12]. When evaluation focuses on standalone protocols, the gathered evidence shows no absolute superiority of one method over the other [13]. Instead, distinct chronological and patient-specific advantages emerge. Daily topical minoxidil 5% continues to serve as a reliable benchmark for long-term structural improvement and hair shaft diameter enhancements [12,15]. Meanwhile, autologous PRP injections offer a faster initial response, evidenced by high early conversion rates to negative hair pull tests within the first 12 weeks, particularly in patients with early vertex involvement (Hamilton-Norwood Stage II) [14].

The available trials suggest a potential clinical advantage of combined multimodal regimens over isolated monotherapies, though these findings must be interpreted in light of study sample sizes and methodological heterogeneity. Pairing topical minoxidil 5% with monthly intradermal PRP infiltrations showed favorable responses across several evaluated parameters, although between-group density differences did not reach statistical significance in all individual trials (e.g., Wei et al., p = 0.26) [13,15]. This synergy operates on a sound biological basis: while minoxidil shortens the telogen phase and promotes vascular supply, the concentrated growth factors in PRP such as PDGF, VEGF, and TGF-beta actively stimulate the proliferation of dermal papilla cells [7]. Recent clinical trial data by Annamreddy et al. further suggest a possible additive effect over a six-month follow-up window, although these single-center findings are limited by a high overall risk of bias [15]. Demonstrating that combined therapy achieves the highest rates of alopecia grade improvement (55%), trichoscopic hair shaft diameter enlargement (66.70%), physician-assessed improvement (>50% score in 85% of cases), and patient self-assessment satisfaction (90%) compared to either minoxidil or PRP monotherapy alone [15]. These findings align with broader systematic reviews, where meta-analytic data by Yao et al. [16] suggest potential hair density gains with additive combined regimens, albeit backed by low-to-very-low certainty of evidence, while broader syntheses comparing standalone PRP against topical minoxidil further contextualize their relative monotherapeutic utility [17]. Additionally, a recent quantitative synthesis by Umar et al. evaluated nine RCTs (n = 451), confirming significant hair density improvements with PRP compared to topical minoxidil [18]. Our review expands upon these quantitative findings by offering an updated qualitative synthesis focused on recent trial protocols, dual-therapy synergistic outcomes (PRP combined with 5% topical minoxidil), and practical clinical feasibility metrics.

From a safety and compliance perspective, both therapeutic tracks present minor localized issues but lack severe systemic complications. Topical minoxidil compliance is mainly limited by epidermal reactions like scalp pruritus, scaling, and hypertrichosis [4,15]. On the other hand, PRP avoids systemic biochemical reactions due to its autologous nature, though procedural discomfort, specifically post-injection pain and transient headache, remains its primary clinical hurdle [12,15]. To mitigate application pain, alternative administration techniques like microneedling have been explored; Nilforoushzadeh et al. demonstrated that microneedling-assisted PRP administration achieves comparable efficacy to direct intradermal injections regarding hair count (88.4% vs. 62.4%) and diameter improvement (51.3% vs. 58.6%, p > 0.05), providing a well-tolerated biostimulatory alternative [19]. While not directly tested as comparative variables within the included trials, broader clinical literature suggests that optimizing protocol standardization (such as double-spin centrifugation parameters) and incorporating patient comfort measures may help improve overall treatment compliance in multimodal protocols.

From a broader contextual standpoint beyond our primary PICO criteria, emerging data on low-dose oral minoxidil (e.g., Álvares Penha et al. [20]) suggests non-superiority in hair density compared to topical 5% formulations alongside higher hypertrichosis rates, further contextualizing topical 5% minoxidil as a primary benchmark.

Limitations

Several methodological limits affect the generalizability of these findings. First, risk of bias evaluation via the Cochrane RoB 2 tool identified an overall high risk of bias in two of the four included RCTs, driven mainly by unblinded, open-label protocols and subjective endpoint reporting. Second, trial procedures showed clear heterogeneity in PRP preparation and delivery, including variations in centrifugation speeds, injection schedules, and total volume administered (3-4 mL). Additionally, primary outcome measures differed across studies, incorporating trichoscopic counts, hair pull test conversions, and subjective self-assessment questionnaires. Lastly, available trial data largely focus on male populations monitored over a six-month window. Furthermore, inherent methodological limitations of this review must be acknowledged: the search window was restricted to studies published from 2021 onward, the final synthesis was based on a small pool of eligible RCTs (n = 4), full-text availability was required for inclusion, and clinical or methodological heterogeneity precluded quantitative meta-analysis and formal GRADE certainty scoring.

## Conclusions

While isolated approaches show distinct clinical merits, with topical minoxidil 5% providing reliable hair shaft diameter improvements over time and plasma biostimulation triggering accelerated early responses, neither agent displays absolute mutual superiority. Combining topical minoxidil with PRP represents a promising therapeutic option that may enhance overall clinical responses in selected patients. Systematically combining local plasma infiltrations with a daily 5% minoxidil regimen may offer more favorable outcomes across specific trichoscopic and patient-reported parameters, although further large-scale, low-bias trials are required to confirm definitive superiority. Ultimately, standardizing these biological preparation parameters and refining procedural pain control stand as primary practical priorities to maximize patient adherence and therapeutic success in clinical practice.
